# Effect of a Topical Nonsteroidal Anti-Inflammatory Drug (0.1% Pranoprofen) on VEGF and COX-2 Expression in Primary Pterygium

**DOI:** 10.3389/fphar.2021.709251

**Published:** 2021-07-09

**Authors:** Bangtao Yao, Fei Wang, Xiaogui Zhao, Bei Wang, Xiaoli Yue, Yuhua Ding, Gang Liu

**Affiliations:** ^1^ Department of Ophthalmology, Nanjing Lishui District People’s Hospital, Lishui Branch of Southeast University Affiliated Zhongda Hospital, Nanjing, China; ^2^ Department of Ophthalmology, Jiangsu Province Hospital, The First Affiliated Hospital of Nanjing Medical University, Nanjing, China; ^3^ Department of Pathology, Nanjing Lishui District People’s Hospital, Lishui Branch of Southeast University Affiliated Zhongda Hospital, Nanjing, China

**Keywords:** 01% Pranoprofen, vascular endothelial growth factor, cyclo-oxygen-ase-2, pterygium, nonsteroidal anti-inflammatory drug

## Abstract

**Purpose:** To evaluate the effect of a topical nonsteroidal anti-inflammatory drug (0.1% pranoprofen) on the expression of VEGF and Cox-2 in primary pterygium.

**Methods:** This was a prospective, randomized study. Between January 2019 and April 2020, 120 patients diagnosed with primary pterygium were enrolled and randomly divided into three groups before operation: 1) 40 patients in group 1 received topical pranoprofen 0.1% four times daily for 4 weeks, 2) 40 patients in group 2 received topical fluorometholone 0.1% four times daily for 4 weeks, and 3) patients in group 3 did not receive treatment. For each group, the age, sex, eye type, best-corrected visual acuity (BCVA), intraocular pressure (IOP), duration of onset, combined systemic diseases, and the results regarding vascular endothelial growth factor (VEGF) and cyclo-oxygen-ase-2 (COX-2) in postoperative pterygial tissues were evaluated in detail.

**Results:** There were no significant differences regarding age, sex, eye type, combined systemic diseases, duration of onset, IOP, and BCVA within the three groups (*p* > 0.05). The reduction of VEGF and CoX-2 expression of pterygial vascular endothelial cells in group 1 were statistically significant compared to group 2 and group 3 (*p* < 0.05). There were significant correlations between COX-2 and VEGF expression of pterygial tissues within the three groups (*p* < 0.05).

**Conclusion:** The present findings suggested that the topical pranoprofen 0.1% could reduce the expression of VEGF and COX-2 in primary pterygium. We confirmed that treatment with pranoprofen offers advantages in early intervention and has therapeutic potential in reducing the postoperative recurrence of primary pterygium patients.

**Clinical Trial registration**: The study was registered with the Chinese Clinical Trial Registry. (http://www.chictr.org.cn/index.aspx, Registration Number: ChiCTR2100047726).

## Introduction

Pterygium is a common, benign conjunctival degenerative disease with a global prevalence, characterized by a proliferative disorder or a neoplastic-like growth lesion in the cornea (Van Acker et al., 2019); its exact pathogenesis is not fully understood (Detorakis and Spandidos, 2009).

There are some existing clinical guidelines for treating primary pterygium. Monitoring is the indicated form of management in the early stages (Guo et al., 2019). Several interventional methods have been proposed in advanced cases, including conjunctival autograft, amniotic membrane, and other adjuvant therapies (Guo et al., 2019). However, no surgical methods can completely prevent the recurrence of pterygium (Clearfield et al., 2017). Therefore, preventing the growth of the early pterygium or reducing its postoperative recurrence is worthy of study.

Recently, some studies have reported that the formation and development of pterygium are associated with vascular endothelial growth factor (VEGF), cyclo-oxygenase (COX), and multiple proinflammatory cytokines (e.g., interleukin-6, interleukin-8) (Adiguzel et al., 2007; Park et al., 2011; Martín-López et al., 2019). Some researchers have used subconjunctival injections of anti-VEGF drugs as adjuvant therapy to treat pterygium and have achieved favourable outcomes (Singh et al., 2015; Mohamed et al., 2018). However, at present, few prospective and comprehensive studies are available on the topical effect of anti-VEGF drugs in patients with primary pterygium.

Pranoprofen is a member of the nonsteroidal anti-inflammatory drug (NSAID) family; it is an inhibitor of the COX enzyme, and has been used to treat pain, fever, and inflammation (Yoshinaga et al., 2011). Previous studies have shown that systemic application of NSAIDs was effective for the treatment of cancer due to its inhibitory effect on VEGF (Ghanghas et al., 2016; Tsoi et al., 2019). Similar outcomes were demonstrated in the treatment of VEGF-related ocular diseases, such as choroidal neovascularization and corneal angiogenesis (Pakneshan et al., 2008; Semeraro et al., 2019a). Recently, topical pranoprofen has been used to treat acute central serous chorioretinopathy and age-related macular degeneration (An and Kwon, 2016; Semeraro et al., 2019b). Fluorometholone is a corticoid, and it can inhibit inflammation by modulating inflammatory mediators such as arachidonic acid metabolites, chemokines, and cytokines, as well as their receptors. Topical fluorometholone has been proven to be safe and effective for patients with primary pterygium (Ozgurhan et al., 2013). However, to the best of our knowledge, the effect of their inhibition of VEGF and Cox-2 on pterygium has not been described in literature. Herein, we evaluated the effects of topical 0.1% pranoprofen, in comparison with fluorometholone, on the expression of VEGF and COX-2 in patients with primary pterygium.

## Materials and Methods

### Study Design

This study was designed as a prospective, randomized study of 120 adult patients diagnosed with primary pterygium in a tertiary general hospital. The patients were enrolled in this study between January 2019 and April 2020. The study was designed and conducted in accordance with the Declaration of Helsinki and was submitted to the appropriate review board of Lishui District People’s Hospital (2019LX01, 22/11/2018). All participating patients provided informed consent for this study. After obtaining an informed consent, the patients were randomly divided into three groups: 1) 40 patients in group 1 received topical pranoprofen 0.1% (Pranopulin; Senju Pharmaceutical Co., Ltd.) four times daily for 4 weeks, 2) 40 patients in group 2 received topical fluorometholone 0.1% (Flumetholon; Santen Pharmaceutical Co., Ltd.) four times daily for 4 weeks, and 3) 40 patients in group 3 did not receive treatment. Randomization was done at the start of the study with prepared randomized digital tables using SPSS software (Version 22.0. IBM Corp., Armonk, NY). An independent observer generated the random allocation sequence and assigned interventions. The allocation sequence was concealed using numbered and sealed opaque envelopes. All other doctors and participants were blinded during and after the assignment.

The patients’ age, sex, eye type, best-corrected visual acuity (BCVA), intraocular pressure (IOP), duration of onset, combined systemic diseases, and the results regarding VEGF and COX-2 expression in postoperative pterygial tissues were recorded. BCVA was converted to the logarithm of the minimum angle of resolution (LogMAR) for data analyses.

Patient follow-up was planned on days 7, 14, 21, and 28 after commencing treatment in groups 1 and 2. All patients were evaluated for any ocular adverse effects at each follow-up, such as burning, pain, or ocular hypertension. Patients with poor compliance or those who could not be followed up on time were withdrawn from the study. Pterygium surgeries (pterygium excision and conjunctival autograft harvest from the supero-temporal bulbar conjunctiva) were performed after 4 weeks of treatment in groups 1 and 2. All the pterygium tissue samples were obtained during the operation and sent to the Department of Pathology for evaluation of VEGF and COX-2 expression. Pterygium was confirmed using haematoxylin and eosin staining.

### Inclusion Criteria

Patients with primary pterygium tissue invading the cornea between 2 and 3 mm from the nasal conjunctiva were enrolled.

### Exclusion Criteria

The exclusion criteria were as follows: 1) pseudopterygium; 2) recurrent pterygium; 3) pterygium with active ocular inflammation; 4) patients who had poor compliance; 5) patients who refused surgery; and 5) the invasion of pterygium tissue was less than 2 mm or more than 3 mm into the cornea.

### Haematoxylin and Eosin staining

Samples from each patient initially underwent haematoxylin and eosin staining. The surgical tissues were fixed in 4% neutral formaldehyde solution for 12 h, and routinely embedded in paraffin, then the tissues were cut into 5 μm slices. The slides were stained with hematoxylin for 8 min and washed several times in running tap water. Then the slides were stained in eosin for 1 min. After that, the slides were dehydrated by placing them consecutively in 80% ethanol, 95% ethanol I and 95% ethanol II solutions for 3 min each time, and they were finally soaked in xylene for 3 min. Air dried slides were mounted with neutral resin and covered with coverslip. Slides were examined by light microscopy.

### Immunohistochemistry

Immunohistochemical staining for VEGF and COX-2 was performed on 4-μm-thick formalin-fixed and paraffin-embedded sections of surgical specimens from pterygium patients using the Elivision™ plus two-step system (BENCHMARK^XT^; Roche, Switzerland). VEGF and COX-2 staining was evaluated in the cytoplasm of vascular endothelial cells. Three slices from each patient and five fields in each slice were analyzed.

### Scoring of Stained Tissue Sections

The expression of VEGF and COX-2 was classified according to the staining intensity and percentage of positively-stained cells. A 4-point rating scale was used to evaluate the staining intensity (0, no staining; 1, weak; 2, moderate; and 3, strong). A 5-point rating scale was used to represent the percentage of positively-stained cells: (0, ≤10%; 1, 11–25%; 2, 26–50%; 3, 51–75%; and 4, > 75%). Finally, a total score (TS) was calculated from the sum of these scores to reflect the expression level: score 0–1, negative (-); score 2–3, weak positive (+); score 4–5, moderate positive (++); and score 6–7, strong positive (+++). The sections were coded and evaluated by two independent experienced pathologists (Dr. Xiaoli Yue and Dr. Ting Zhang), who were blinded to the patients’ data. If discrepancies occurred, a third investigator (Dr. Hongbing Xiong) evaluated the tissue sections to obtain consistent results and a consensus was reached among the three investigators. The TS and expression levels of VEGF and COX-2 in the tissue sections of the three groups were statistically observed, and the differences between the three groups were analyzed.

### Statistical Analysis

Data from the patients’ clinical records were processed with SPSS (Statistical Package for Social Sciences, version 23.0, IBM). Continuous variables were expressed as mean ± standard deviation, and categorical variables were expressed as counts (%). For categorical variables, Pearson’s χ2 test was used; for continuous variables, the Kruskal–Wallis test was used. Relationships between VEGF and COX-2 expression were assessed using Spearman’s correlation analysis. Two-tailed tests of significance were performed, and *p*-values <0.05 were considered statistically significant.

## Results

### Demographics

A total of 120 patients diagnosed with primary pterygium were randomly divided into three groups. There was a loss of two patients in group 1 and three patients in group 2 during the study period because two patients moved to different cities and three patients did not comply with the surgery and follow-up schedule. Therefore, the data of 115 patients with primary pterygium were included in the final analysis.

In the final 115 patients, 52 were male and 63 were female; their average age was 64.21 ± 8.53 years (range 45–85). Primary pterygium occurred in the right eye in 67 patients, and in the left eye in 48 patients. Group 1 (n = 38) comprised 18 (47.4%) men and 20 (52.6%) women, with an average age of 64.32 ± 9.22 years. It included 20 (52.6%) and 18 (47.4%) cases of primary pterygium in the right eye and left eye, respectively. The median duration of onset was 9.95 years, and 11 (28.9%) cases were combined with systemic diseases. Group 2 (n = 37), comprised 15 (40.5%) men and 22 (59.5%) women with an average age of 64.78 ± 8.11 years. It included 21 (56.8%) and 16 (43.2%) cases of primary pterygium in the right eye and left eye, respectively. The median duration of onset was 9.22 years, and 10 cases (27.0%) were combined with systemic diseases. Group 3 (n = 40) comprised 19 (47.5%) men and 21 (52.5%) women with an average age of 63.57 ± 8.39 years. It included 26 (65.0%) and 14 (35.0%) cases of primary pterygium in the right eye and left eye, respectively. The median duration of onset was 8.5 years, and 13 (32.5%) cases were combined with systemic diseases (Table 1). No obvious ocular side effects were observed in our study.

**TABLE 1:** Demographic and clinical characteristics of the study participants.

	Group 1	Group 2	Group 3	*p*-value
Eyes, n	38	37	40	—
Sex, n (%)	—	—	—	0.786
Male	18 (47.4)	15 (40.5)	19 (47.5)	
Female	20 (52.6)	22 (59.5)	21 (52.5)	
Age, years (mean ± SD)	64.32 ± 9.22	64.78 ± 8.11	63.57 ± 8.39	0.381
Eye type, n (%)	—	—	—	0.528
Right	20 (52.6)	21 (56.8)	26 (65.0)	
Left	18 (47.4)	16 (43.2)	14 (35.0)	
Duration of onset, years	9.95 (5.75–10.0)	9.22 (5.0–10.0)	8.50(5.0–10.0)	0.703
Combined systemic disease, n (%)	11 (28.9)	10 (27.0)	13 (32.5)	0.866
LogMAR BCVA (mean ± SD)	0.41 ± 0.24	0.44 ± 0.25	0.44 ± 0.27	0.778
Intraocular pressure, mmHg (mean ± SD)	14.34 ± 1.94	14.76 ± 2.21	13.85 ± 1.89	0.146
VEGF, median (range)	4 (3–4)	4 (4–5)	5 (4–5)	0.001
Negative (-), n (%)	6 (15.8%)	0	0	
Weak positive (+), n (%)	7 (18.4%)	8 (21.6%)	7 (17.5%)	—
Moderate positive (++), n (%)	25 (65.8%)	24 (64.9%)	28 (70.0%)	—
Strong positive (+++), n (%)	0	5 (13.5%)	5 (12.5%)	—
COX-2, median (range)	3 (2–4)	4 (3–5)	4 (4–5)	0.003
Negative (−), n (%)	8 (21.1%)	1 (2.7%)	1 (2.5%)	—
Weak positive (+), n (%)	14 (36.8%)	12 (32.4%)	4 (10.0%)	—
Moderate positive (++), n (%)	16 (42.1%)	20 (54.1%)	32 (80.0%)	—
Strong positive (+++), n (%)	0	4 (10.8%)	3 (7.5%)	—

VEGF: vascular endothelial growth factor; COX: cyclo-oxygen-ase; LogMAR BCVA: logarithm of the minimum angle of resolution best-corrected visual acuity.

### Post-Surgical Evaluation of Clinical Outcomes

The suture was removed at the 7-days follow-up after the operation. The cornea was completely repaired within 10 days, and the conjunctival wound was not cracked or curled. There were no postoperative complications such as graft detachment, corneal ulcer, or formation of granulomas. At the 9-months follow-up, there was recurrence in 2 (5.3%), 2 (5.4%), and 3 (7.5%) cases in groups 1, 2, and 3, respectively (all *p* > 0.05, not showed in the table). The recurrence was defined by any new fibrovascular tissue crossing the limbus and extending over the cornea.

### Combined Systemic Diseases

In group 1, 10 cases were complicated with hypertension, and one was complicated with diabetes mellitus. In group 2, there were eight and two cases complicated with hypertension and diabetes mellitus, respectively. In group 3, there were 11 and two cases complicated with hypertension and diabetes mellitus, respectively (Table 1).

### Haematoxylin and Eosin staining

**FIGURE 1:**
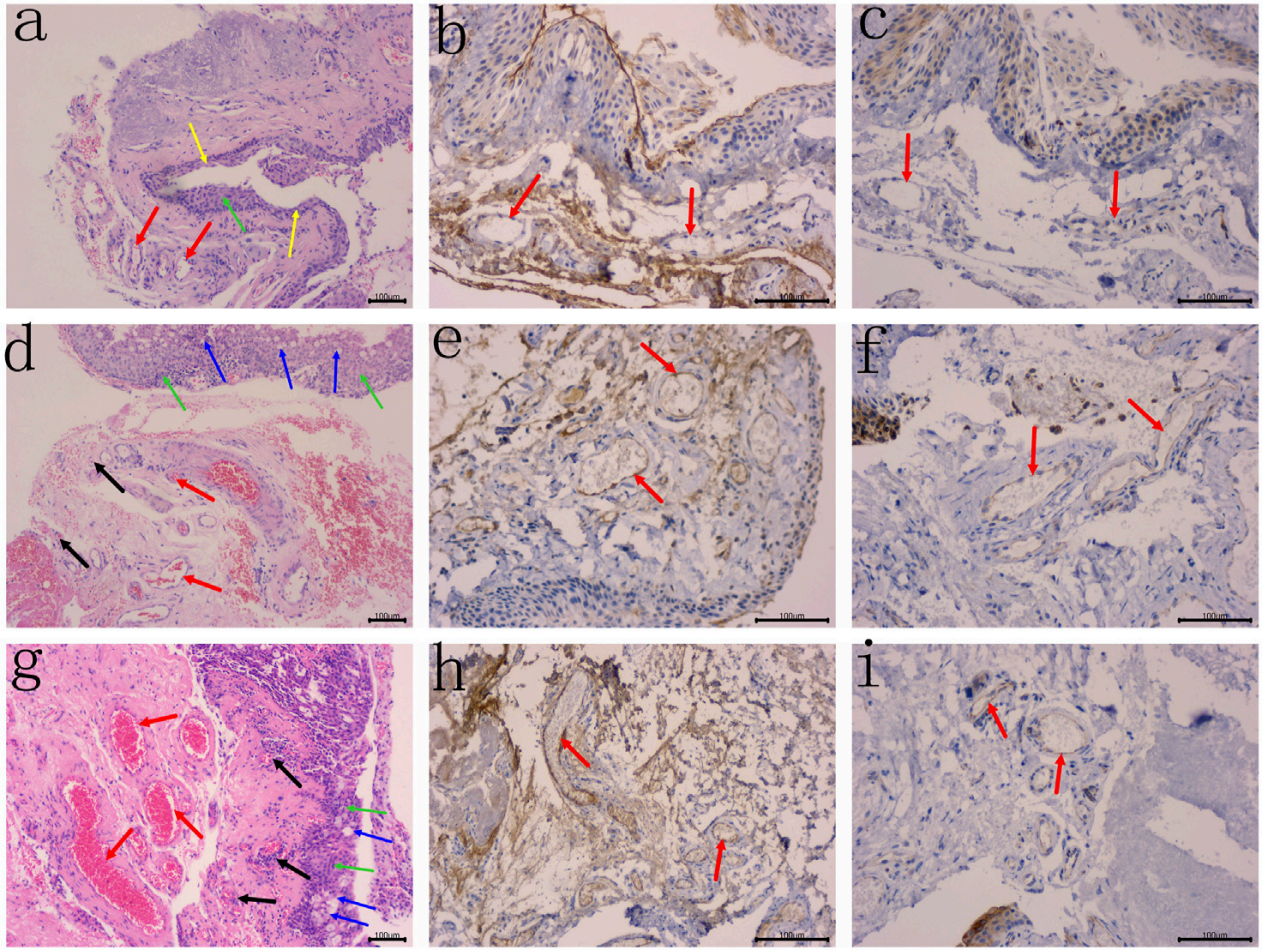
The atrophy of epithelial cells (yellow arrows) and/or hyperplasia (green arrows), with the presence of increased goblet cells (blue arrows) were noted, distinct fibroblast proliferation with mild inflammatory cell infiltration were observed under the mucosa. **(A)** HE staining of human primary pterygium (case 15 in group 1) is demonstrated, the small vessels (red arrows) were observed, without evidence of capillary hyperplasia and vasodilation, original magnification: 100×. **(B–C)** Primary pterygium specimen with negative VEGF and COX-2 expression, respectively. No staining reaction in the cytoplasm of vascular enothelial cells (red arrows), original magnification: **B–C** 200×. **(D)** HE staining of human primary pterygium (case 9 in group 2) is demonstrated, moderate capillary hyperplasia indicating angiogenesis (black arrows), and moderate vasodilation (red arrows) were noted, original magnification: 100×. **(E–F)** Primary pterygium specimen with moderate VEGF and COX-2 expression, respectively. Note the typical diffuse staining reaction in the cytoplasm of vascular endothelial cells (red arrows), original magnification: **E–F** 200×. **(G)** HE staining of human primary pterygium (case 37 in group 3) is demonstrated, capillary cluster hyperplasia indicating severe angiogenesis (black arrows), and severe vasodilation (red arrows) were observed, original magnification: 100×. **(H–I)** Primary pterygium specimen with high VEGF and COX-2 expression, respectively. Note the typical diffuse staining reaction in the cytoplasm of vascular endothelial cells (red arrows), original magnification: **H–I** 200×.

All the haematoxylin and eosin staining slides can be characterized by the atrophy and/or hyperplasia of epithelial cells, with the presence of increased goblet cells, distinct fibroblast proliferation with mild inflammatory cell infiltration were observed under the mucosa, the capillary hyperplasia and vasodilation were also noted (Figures 1A,D,G). These evidences were consistent with the pathological findings of pterygium.

### VEGF

The VEGF expression levels in the pterygium are listed in Tables 1 and 2. In group 1, there were 6, 7, 19, and 6 samples with a TS of 0, 3, 4, and 5, respectively. Six (15.8%), 7 (18.4%), and 25 (65.8%) samples were classified as negative (-), weak positive (+), and moderate positive (++), respectively. No samples were classified as strong positive (+++). In group 2, there were 1, 7, 13, 11, and 4 samples with a TS of 2, 3, 4, 5, and 6, respectively. One sample had a TS of 7. Eight (21.6%), 24 (64.9%), and 5 (13.5%) samples were classified as weak positive (+), moderate positive (++), and strong positive (+++), respectively. No sample was classified as negative (-). In group 3, there were 1, 6, 9, 19, and 3 samples with a TS of 2, 3, 4, 5, and 6, respectively. Two samples had a TS of 7. Seven (17.5%), 28 (70.0%), and 5 (12.5%) samples were classified as weak positive (+), moderate positive (++), and strong positive (+++), respectively. No sample was classified as negative (-) (Figures 1B, E, H) (Tables 1, 2).

**TABLE 2:** VEGF and COX-2 expression in pterygial vascular endothelial cells.

Group	No	Total score		Expression level			
		VEGF, median (range)	COX-2, median (range)	VEGF, n (%)		COX-2, n (%)	
				Negative	Positive	Negative	Positive
1	38	4 (3–4)	3 (2–4)	6 (15.8%)	32 (84.2%)	8 (21.1%)	30 (78.9%)
2	37	4 (4–5)	4 (3–5)	0	37 (100%)	1 (2.7%)	36 (97.3%)
3	40	5 (4–5)	4 (4–5)	0	40 (100%)	1 (2.5%)	39 (97.5%)
*p*-value		1 VS 2: 0.024	1 VS 2: 0.018	1 VS 2: 0.036		1 VS 2: 0.037
		1 VS 3: 0.001	1 VS 3: 0.005	1 VS 3: 0.028		1 VS 3: 0.027	
		2 VS 3: 0.886	2 VS 3: 1.000	2 VS 3: -		2 VS 3: 1.000	

VEGF: vascular endothelial growth factor; COX: cyclo-oxygen-ase.

### COX-2

The COX-2 expression levels in pterygium studied are listed in Tables 1, 2. In group 1, there were 3, 5, 4, 10, 9, and 7 samples with a TS of 0, 1, 2, 3, 4, and 5, respectively. Eight (21.1%), 14 (36.8%), and 16 (42.1%) samples were classified as negative (-), weak positive (+), and moderate positive (++), respectively. No samples were classified as strong positive (+++). In group 2, there were 1, 5, 7, 8, 12, and 4 samples with a TS of 1, 2, 3, 4, 5, and 6, respectively. One (2.7%), 12 (32.4%), 20 (54.1%), and 4 (10.8%) samples were classified as negative (-), weak positive (+), moderate positive (++), and strong positive (+++). In group 3, there were 1, 3, 1, 23, 9, and 3 samples with a TS of 1, 2, 3, 4, 5, and 6, respectively. One (2.5%), 4 (10.0%), 32 (80.0%), and 3 (7.5%) samples were classified as negative (-), weak positive (+), moderate positive (++), and strong positive (+++), respectively (Figure 1) (Tables 1, 2).

### Correlations Between TS and VEGF and COX-2 Expression

**FIGURE 2:**
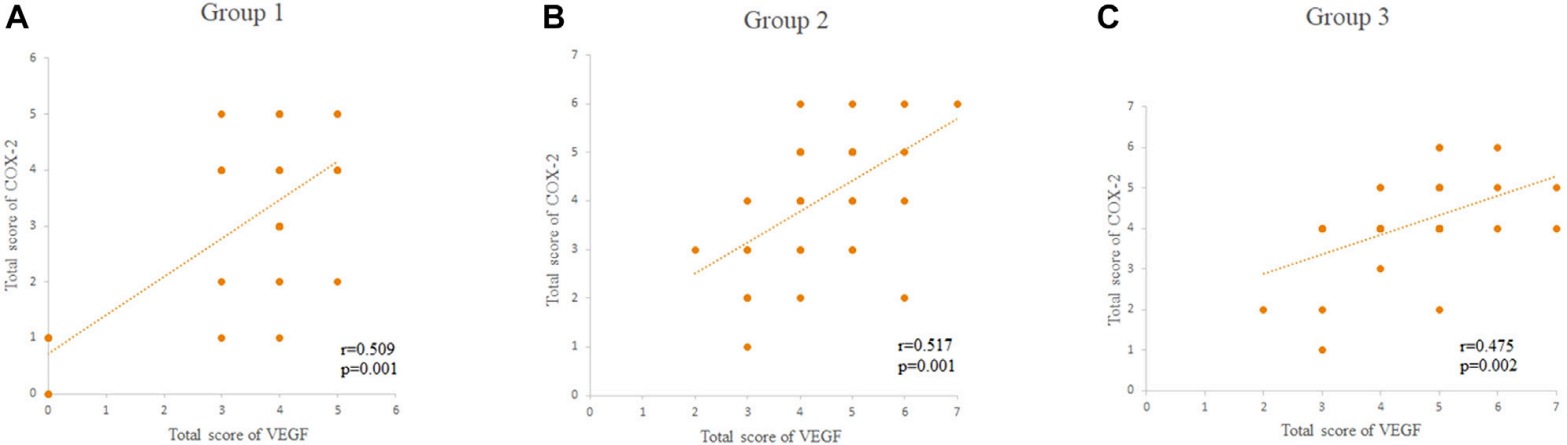
**(A–C)** The correlations between the total score of VEGF and COX-2 within the groups 1, 2, and 3, respectively. The total score of VEGF and COX-2 was scored as 0–7. HE: Hematoxylin and eosin; VEGF: vascular endothelial growth factor; COX-2: cyclo-oxygen-ase-2.

There were significant correlations between the TS and expression of VEGF and COX-2 in pterygial vascular endothelial cells within groups 1, 2, and 3 (all *p* < 0.05, Spearman’s coefficient of correlation), as shown in Figure 2 and Table 3, respectively.

**TABLE 3:** Correlations between levels of VEGF and COX-2 expression in primary pterygium patients.

Group No		—	COX-2^a^				r	*p*-value
1	38	VEGF^a^	Negative (−)	Weak positive (+)	Moderate positive (++)	Strong positive (+++)	0.473	0.003
		Negative (−)	6	0	0	0		
		Weak positive (+)	1	2	4	0		
		Moderate positive (++)	1	12	12	0		
		Strong positive (+++)	0	0	0	0		
2	37	VEGF^a^	—	—	—	—	0.550	<0.001
		Negative (−)	0	0	0	0		
		Weak positive (+)	1	6	1	0		
		Moderate positive (++)	0	5	17	2		
		Strong positive (+++)	0	1	2	2		
3	40	VEGF^a^	—	—	—	—	0.413	0.008
		Negative (−)	0	0	0	0		
		Weak positive (+)	1	2	4	0		
		Moderate positive (++)	0	2	24	2		
		Strong positive (+++)	0	0	4	1		

a Detection of the expressing level of VEGF and COX-2 by immunohistochemistry.

### Group Comparisons

There were no significant differences in age, sex, eye type, combined systemic diseases, duration of the disease, IOP, and BCVA among the three groups (all *p* > 0.05). The TS and expression levels of VEGF and COX-2 in pterygium tissues in group 1 were significantly lower than those in groups 2 and 3 (all *p* < 0.05). However, there were no significant differences in TS and expression levels of VEGF and COX-2 between groups 3 and 2 (all *p* > 0.05) (Tables 1, 2).

## Discussion

Pterygium is a common, benign conjunctival degenerative disease, which has a widespread incidence (Van Acker et al., 2019). While the underlying mechanism remains unclear, it is generally assumed that it is associated with exposure to ultraviolet irradiation (Detorakis and Spandidos, 2009). In our study, we evaluated the effect of topical 0.1% pranoprofen on the expression of VEGF and Cox-2 in 38 primary pterygium tissues. Our results revealed that VEGF and COX-2 expression was increased in vascular endothelial cells in group 3, which demonstrated that they may be part of the critical pathophysiological mechanism of primary pterygium. Compared to group 2 and group 3, the expression of VEGF and COX-2 in group 1 was significantly decreased, indicating that the application of 0.1% pranoprofen inhibited VEGF and COX-2 expression.

The formation and development of cancer has been shown to be closely associated with the overexpression of VEGF (Padera et al., 2008). Similarly, the pterygium presents tumour-like features (Chui et al., 2011), and recent evidence has indicated that the formation and development of pterygium are probably related to increased expression of VEGF, COX, and multiple proinflammatory cytokines (Martín-López et al., 2019; Adiguzel et al., 2007; Park et al., 2011). Wu et al. (2017) found that long-term exposure to ultraviolet radiation caused the upregulation of these factors, which may result in pterygium. VEGF was demonstrated to be the most potent factor (Detorakis and Spandidos, 2009). Fukuhara et al. (2013) showed that VEGF expression was increased in the vascular endothelial component of pterygium compared with the normal conjunction.

The beneficial effects of anti-VEGF therapies in the treatment of primary pterygium have been widely reported. Sarac et al. (2014), Singh et al. (2015) have reported the regression of pterygial tissues and corneal neovascularization in patients with pterygium who received bevacizumab injections into the pterygial tissue. Nava Castaneda et al. (2014) also reported a lower recurrence rate after adjuvant subconjunctival bevacizumab injection to the conjunctival autograft for primary pterygium. Kasetsuwan et al. (2015) investigated the use of topical bevacizumab after pterygium excision and reported similar beneficial effects. However, Nava-Castaneda et al. (2014) found conjunctival autograft ischemia was a common complication after subconjunctival bevacizumab injections. In addition, anti-VEGF therapies are relatively expensive, invasive, and must be administered frequently.

Recently, NSAIDs have been proven to be potent for the treatment of cancer due to their inhibitory effect on VEGF and COX-2 (Ghanghas et al., 2016; Tsoi et al., 2019). Similarly, reports have demonstrated that NSAIDs are effective in suppressing the retinal and corneal angiogenic response to VEGF (Pakneshan et al., 2008; Semeraro et al., 2019a), and nonselective NSAIDs have been shown to suppress VEGF-induced angiogenesis better than selective COX inhibitors (Singer et al., 2015). However, the exact mechanism by which NSAIDs inhibit VEGF remains unknown. Yoshinaga et al. (2011) proposed that NSAIDs inhibit VEGF-related choroidal neovascularization through the HO-1-dependent pathway, while Pakneshan et al. (2008) proposed that NSAIDs suppress the expression of VEGF by inhibiting the COX enzyme.

Pranopulin is a 0.1% pranoprofen topical ophthalmic agent. Topical pranoprofen non-selectively inhibits the expression of COX-1 and COX-2, suppresses the bio-activity of prostaglandins, and is used to alleviate ocular inflammation, cystoid macular oedema, and postoperative uncomfortable symptoms (Akyol-Salman et al., 2007; Zhu et al., 2018). Recent studies have found that 0.1% pranoprofen was well tolerated and benefited patients with dry eye (Chen et al., 2014). Compared with 0.1% fluorometholone, pranoprofen reduced the IOP level and indicated better anti-inflammatory efficacy and safety (Li et al., 2013). Topical pranoprofen has been proven effective for acute central serous chorioretinopathy and age-related macular degeneration (An and Kwon, 2016; Semeraro et al., 2019b).

Based on the above findings, we assumed that the application of a topical nonsteroidal anti-inflammatory drug (0.1% pranoprofen) may be effective and safe for pterygium. Postoperative recurrence of pterygium patients has been shown to be related to the levels of VEGF and inflammatory factors (Adiguzel et al., 2007). In the present study, we did not observe any difference in postoperative recurrence between the three groups in the 9-months follow-up period, and we concluded that this was due to the following reasons. First, the follow-up duration in our study may not be sufficient to evaluate the efficacy of our interventions with respect to the recurrence. We consider that recurrence would be lower in group 1 than in the two other groups in the long term follow-up. Second, the recurrent and total sample size in each group was small. More data from multiple centers and longer-term follow-up are required to better understand the effect of topical 0.1% pranoprofen on primary pterygium. No obvious ocular side effects were observed in our study, suggesting that the treatments in groups 1 and 2 were safe and well-tolerated.

Glucocorticoids have been shown to suppress the expression of several inflammation-related factors such as arachidonic acid and COX-2, and are used for their anti-inflammatory effect (Lim et al., 2014). In group 2, the patients were treated with topical 0.1% fluorometholone; however, there were no significant differences in the expression of VEGF and COX-2 between groups 2 and 3. This suggests that topical 0.1% fluorometholone cannot downregulate the expression of COX-2. A previous study of nasal polyps by Pujols et al. (2009) has reported similar findings. As a result, we suggest that the true effect of glucocorticoids on COX-2 in the pterygium tissues may be unclear.

Our analysis of the correlations between the TS and VEGF and COX-2 expression within the three groups found that Cox-2 and VEGF expression was closely correlated in the pterygium tissues, which is consistent with the discovery of Liu D et al. (2017). VEGF and COX-2 were frequently found at the basal and middle layers of the endothelial cells of the microvessels. It also demonstrated that the over-expression of COX-2 is associated with increased levels of angiogenic factors, including VEGF in cultured human umbilical vein endothelial cells (Koolwijk et al., 1996). Therefore, we concluded that inflammatory factors (e.g., interleukin-6, interleukin-8, and prostaglandin) may have a significant role in the pathogenesis of vascular permeability, which can induce the expression of VEGF. Similarly, VEGF may stimulate COX-2 expression in pterygial vascular endothelial cells.

There were several limitations to this study. First, it had a small sample size. Second, the follow-up time was relatively short. Third, for ethical reasons, normal conjunctival tissue could not be obtained during the operation. Fourth, it is unknown whether 0.1% pranoprofen is effective for recurrent pterygium.

## Conclusion

We obtained data on the effect of 0.1% pranoprofen on the expression of VEGF and COX-2 in primary pterygium tissue, providing an evidence-based basis for the early treatment of pterygium. We found that VEGF and COX-2 expression was closely correlated in the pterygium tissues. In other words, we confirmed that treatment with pranoprofen offers advantages in early intervention and has therapeutic potential in reducing the postoperative recurrence of primary pterygium patients.

## Data Availability

The original contributions presented in the study are included in the article/supplementary material, further inquiries can be directed to the corresponding authors.

## Abbreviations

BCVA, best-corrected visual acuity; COX, cyclo-oxygen-ase; IOP, intraocular pressure; LogMAR, logarithm of the minimum angle of resolution; NSAID, nonsteroidal anti-inflammatory drug; TS, total score; VEGF, vascular endothelial growth factor.
