# Corrigendum: Effect of a Topical Nonsteroidal Anti-Inflammatory Drug (0.1% Pranoprofen) on VEGF and COX-2 Expression in Primary Pterygium

**DOI:** 10.3389/fphar.2021.743733

**Published:** 2021-08-13

**Authors:** Bangtao Yao, Fei Wang, Xiaogui Zhao, Bei Wang, Xiaoli Yue, Yuhua Ding, Gang Liu

**Affiliations:** ^1^ Department of Ophthalmology, Nanjing Lishui District People’s Hospital, Lishui Branch of Southeast University Affifiliated Zhongda Hospital, Nanjing, China; ^2^ Department of Ophthalmology, Jiangsu Province Hospital, The First Affifiliated Hospital of Nanjing Medical University, Nanjing, China; ^3^ Department of Pathology, Nanjing Lishui District People’s Hospital, Lishui Branch of Southeast University Affifiliated Zhongda Hospital, Nanjing, China

**Keywords:** 0.1% pranoprofen, vascular endothelial growth factor, cyclo-oxygen-ase-2, pterygium, nonsteroidal anti-inflammatory drug

In the original article, there was a mistake in Table 3 as published. Some numbers were wrongly reported in the COX-2, r, and *p*-value columns. “Additionally, there were multiple errors in the Results section due to incorrectly reported or missing values, which have now been corrected or added into the article.” The corrected Table 3 appears below.

**TABLE 3:** Correlations between levels of VEGF and COX-2 expression in primary pterygium patients.

Group No		—	COX-2^a^				r	*p*-value
1	38	VEGF^a^	Negative (−)	Weak positive (+)	Moderate positive (++)	Strong positive (+++)	0.473	0.003
		Negative (−)	6	0	0	0		
		Weak positive (+)	1	2	4	0		
		Moderate positive (++)	1	12	12	0		
		Strong positive (+++)	0	0	0	0		
2	37	VEGF^a^	—	—	—	—	0.550	<0.001
		Negative (−)	0	0	0	0		
		Weak positive (+)	1	6	1	0		
		Moderate positive (++)	0	5	17	2		
		Strong positive (+++)	0	1	2	2		
3	40	VEGF^a^	—	—	—	—	0.413	0.008
		Negative (−)	0	0	0	0		
		Weak positive (+)	1	2	4	0		
		Moderate positive (++)	0	2	24	2		
		Strong positive (+++)	0	0	4	1		

a Detection of the expressing level of VEGF and COX-2 by immunohistochemistry.

A correction has been made to the Results section, sub-section VEGF:

“The VEGF expression levels in the pterygium are listed in Tables 1 and 2. In group 1, there were 6, 7, 19, and 6 samples with a TS of 0, 3, 4, and 5, respectively. Six (15.8%), 7 (18.4%), and 25 (65.8%) samples were classified as negative (-), weak positive (+), and moderate positive (++), respectively. No samples were classified as strong positive (+++). In group 2, there were 1, 7, 13, 11, and 4 samples with a TS of 2, 3, 4, 5, and 6, respectively. One sample had a TS of 7. Eight (21.6%), 24 (64.9%), and 5 (13.5%) samples were classified as weak positive (+), moderate positive (++), and strong positive (+++), respectively. No sample was classified as negative (-). In group 3, there were 1, 6, 9, 19, and 3 samples with a TS of 2, 3, 4, 5, and 6, respectively. Two samples had a TS of 7. Seven (17.5%), 28 (70.0%), and 5 (12.5%) samples were classified as weak positive (+), moderate positive (++), and strong positive (+++), respectively. No sample was classified as negative (-) (Figures 1B,E,H) (Tables 1, 2).”

A correction has been made to the Results section, sub-section COX-2: 

“The COX-2 expression levels in pterygium studied are listed in Tables 1, 2. In group 1, there were 3, 5, 4, 10, 9, and 7 samples with a TS of 0, 1, 2, 3, 4, and 5, respectively. Eight (21.1%), 14 (36.8%), and 16 (42.1%) samples were classified as negative (-), weak positive (+), and moderate positive (++), respectively. No samples were classified as strong positive (+++). In group 2, there were 1, 5, 7, 8, 12, and 4 samples with a TS of 1, 2, 3, 4, 5, and 6, respectively. One (2.7%), 12 (32.4%), 20 (54.1%), and 4 (10.8%) samples were classified as negative (-), weak positive (+), moderate positive (++), and strong positive (+++). In group 3, there were 1, 3, 1, 23, 9, and 3 samples with a TS of 1, 2, 3, 4, 5, and 6, respectively. One (2.5%), 4 (10.0%), 32 (80.0%), and 3 (7.5%) samples were classified as negative (-), weak positive (+), moderate positive (++), and strong positive (+++), respectively (Figure 1) (Tables 1, 2).”

A correction has been made to the Results section, sub-section Correlations Between TS and VEGF and COX-2 Expression:

“There were significant correlations between the TS and expression of VEGF and COX-2 in pterygial vascular endothelial cells within groups 1, 2, and 3 (all P < 0.05, Spearman's coefficient of correlation), as shown in Figure 2 and Table 3, respectively.”

Lastly, a correction has been made to the Results section, sub-section Group Comparisons:

“There were no significant differences in age, sex, eye type, combined systemic diseases, duration of the disease, IOP, and BCVA among the three groups (all p > 0.05). The TS and expression levels of VEGF and COX-2 in pterygium tissues in group 1 were significantly lower than those in groups 2 and 3 (all p < 0.05). However, there were no significant differences in TS and expression levels of VEGF and COX-2 between groups 3 and 2 (all P > 0.05) (Tables 1, 2).”

The authors apologize for this error and state that this does not change the scientific conclusions of the article in any way. The original article has been updated.

